# Perihilar Extrahepatic Cholangiocarcinoma With Typical and Atypical Manifestations: Diagnostic Resolution During Autopsy

**DOI:** 10.7759/cureus.103879

**Published:** 2026-02-18

**Authors:** Javier A Teco-Cortes, Tania A Galindo-García, Paola X Calderón-Navarro

**Affiliations:** 1 Department of Pathology, Instituto Nacional de Ciencias Médicas y Nutrición "Salvador Zubirán", México City, MEX

**Keywords:** acute pancreatitis, autopsy, cholangiocarcinoma, extrahepatic bile ducts, immunohistochemistry

## Abstract

Extrahepatic cholangiocarcinoma is a rare malignant neoplasm characterized by aggressive behavior and poor prognosis. It typically presents in the seventh to eighth decades of life, with a slight male predominance, and most commonly manifests with symptoms related to biliary obstruction; however, atypical clinical presentations may occur. We report the case of a 71-year-old man who presented with acute pancreatitis. Imaging studies failed to identify an underlying neoplasm, and the diagnosis was established only during the autopsy, which revealed a perihilar extrahepatic cholangiocarcinoma involving the proximal extrahepatic bile ducts, with extensive multiorgan infiltration. These findings accounted for the patient’s atypical manifestations, including renal dysfunction and gastrointestinal bleeding, which were fully elucidated through postmortem examination. Extrahepatic cholangiocarcinoma represents a diagnostic challenge due to its nonspecific clinical features and its anatomically difficult location for biopsy, particularly when atypical manifestations predominate. This case highlights the value of autopsy in clarifying complex clinicopathologic correlations and provides observations regarding the role of immunohistochemistry in the diagnosis, prognostic assessment, and therapeutic management of these tumors.

## Introduction

Carcinoma of the extrahepatic bile ducts (EHBD) is a malignant epithelial neoplasm arising from the extrahepatic biliary tree. It most commonly occurs during the seventh and eighth decades of life, with a slight male predominance, and frequently presents with obstructive jaundice [1]. Its incidence ranges from fewer than 0.2 to more than 2.24 cases per 100,000 person-years worldwide, with higher prevalence in Asian countries [2]. Risk factors are largely associated with chronic inflammation, including primary sclerosing cholangitis, biliary cysts, Caroli disease, and cholelithiasis. Endemic parasites (in particular, *Opisthorchis viverrini* and *Clonorchis sinensis*) are the primary etiological factors in endemic regions. Four recognized precursor lesions have been described: biliary intraepithelial neoplasia, intraductal papillary neoplasm, intraductal oncocytic papillary neoplasm, and intraductal tubulopapillary neoplasm [1,3].

Macroscopically, extrahepatic cholangiocarcinoma may exhibit sclerosing, nodular, or papillary growth patterns, with the sclerosing type being the most common, characterized by annular constrictive thickening of the bile ducts. Microscopically, a wide range of morphologic patterns has been reported, most frequently adenocarcinoma with a pancreatobiliary phenotype, accompanied by a prominent desmoplastic reaction, lymphovascular invasion, and perineural invasion [4].

The most important prognostic factor is tumor stage at diagnosis, as well as resectability. Five-year overall survival rates range from 11% to 31% in resectable cases, whereas in unresectable disease, survival approaches 0% [1,4].

## Case presentation

A 71-year-old man with no significant past medical history initially presented with epigastric abdominal pain and multiple episodes of food-content vomiting. He was diagnosed with intestinal obstruction, which was managed with enemas without improvement. A non-contrast abdominal CT scan was subsequently performed, revealing Balthazar grade D pancreatitis. Medical management was initiated, with no significant clinical improvement.

One month later, magnetic resonance cholangiopancreatography (MRCP) demonstrated acute inflammatory changes in the pancreas, biliary sludge, dilation of the proximal right collecting system, and bilateral pleural effusions, without evidence of intrahepatic biliary dilation, ductal wall thickening, or stricturing, prompting hospital admission. Laboratory studies showed metabolic acidosis and liver function abnormalities (Table 1).

**Table 1:** Laboratory test results

Parameters	Patient Value	Unit	Reference Range (Adults)
Serum Creatinine	6.5	mg/dL	0.6–1.3
Blood Urea Nitrogen (BUN)	119	mg/dL	7–20
Total Bilirubin	9.8	mg/dL	0.1–1.2
Alanine Aminotransferase (ALT)	171	U/L	7–56
Aspartate Aminotransferase (AST)	363	U/L	10–40
Alkaline Phosphatase (ALP)	709	U/L	44–147

A triphasic pancreatic CT scan demonstrated acute pancreatitis with >30% necrosis, biliary tract enhancement compatible with cholangitis without biliary or pancreatic duct dilation, right-sided hydronephrosis due to right ureteral obstruction, bilateral pleural effusions, and ascites. Endoscopic retrograde cholangiopancreatography was attempted, but biliary cannulation was unsuccessful.

During the procedure, the patient developed hemodynamic instability with atrial flutter with rapid ventricular response, requiring cardioversion, amiodarone, and vasopressor support. He subsequently developed worsening of renal function, necessitating renal replacement therapy with hemodialysis, along with respiratory deterioration and lower gastrointestinal bleeding. Despite aggressive management, he progressed to irreversible hemodynamic collapse and died. The final clinical diagnosis was severe necrotizing acute pancreatitis and hypovolemic shock.

Postmortem pathological findings

**Figure 1:**
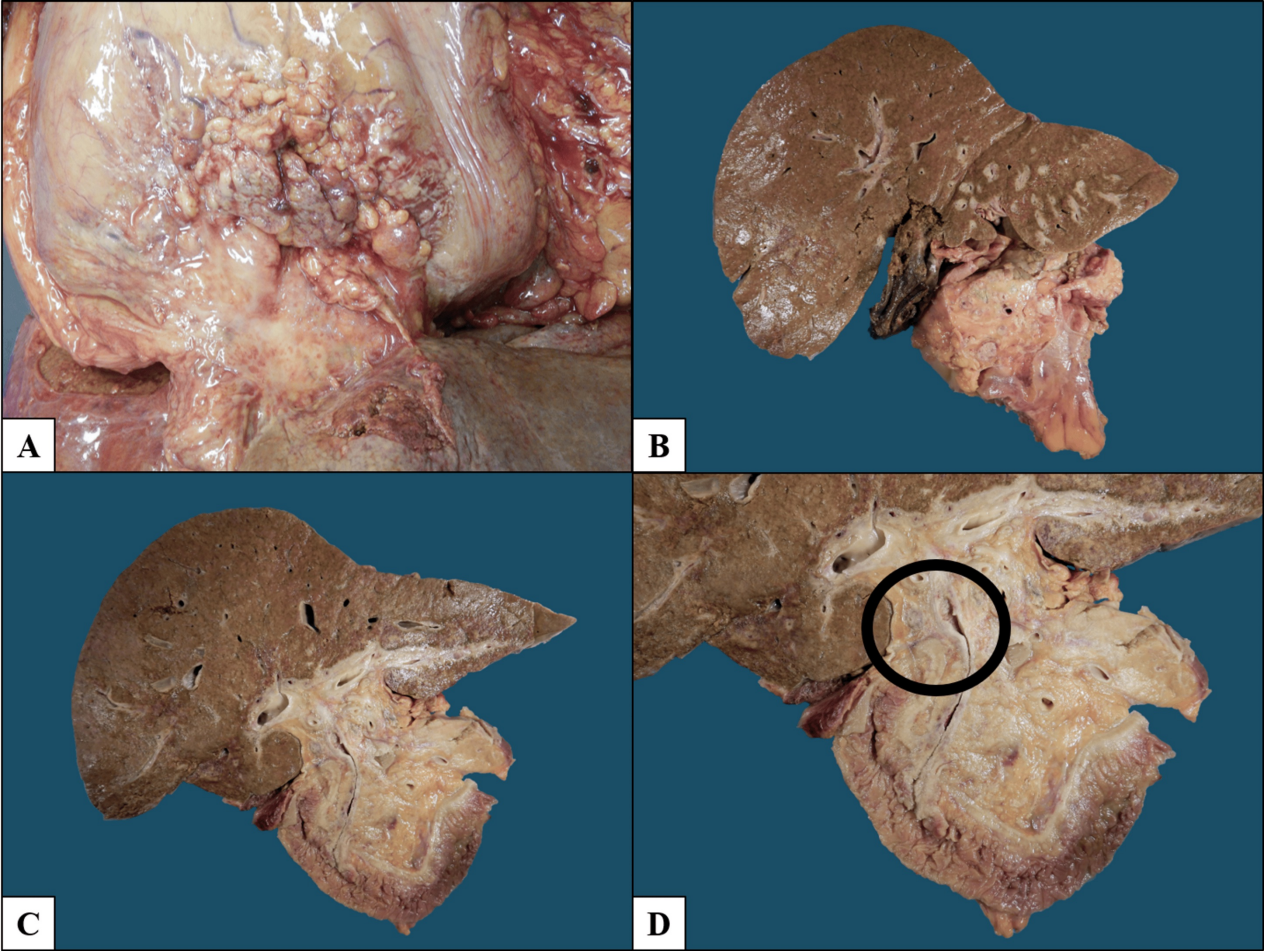
Gross findings. (A) Diffuse peritoneal carcinomatosis with multiple adhesions among abdominal organs. (B) Pancreas is distorted by a poorly defined infiltrative lesion, while the liver shows multiple lesions surrounding the bile ducts; the gallbladder is grossly unremarkable. (C) Serial sectioning demonstrates that the lesion involving the liver and pancreas originates from the extrahepatic bile ducts. (D) Close-up view of the primary tumor arising from the cystic duct and common hepatic duct, showing concentric wall thickening consistent with a sclerosing growth pattern.

An autopsy was performed, revealing a male phenotype with generalized jaundice. Upon opening the body cavities, bilateral pleural effusions, ascites, and diffuse peritoneal carcinomatosis were identified, with multiple adhesions among abdominal organs, particularly involving the stomach, pancreas, small intestine, and colon. Dissection revealed a primary neoplasm arising in the extrahepatic bile ducts, specifically involving the cystic duct and common hepatic duct, with a sclerosing gross appearance (Figure 1).

The tumor showed diffuse infiltration of the pancreas and liver, extension into the walls of hollow viscera, and spread through abdominal soft tissues encasing the descending aorta, inferior vena cava, and bilateral renal vessels, extending to both renal hila.

Multiple peritracheal, bilateral peribronchial, and abdominal lymph nodes were identified. The thyroid gland appeared pale and grayish with a smooth cut surface and no grossly apparent lesions. The right adrenal gland showed a well-defined, ovoid hemorrhagic lesion measuring 4.0 × 3.0 cm, replacing nearly the entire gland, whereas the contralateral adrenal showed only hemorrhagic changes of the medulla. The lungs, heart, spleen, ureters, and bladder showed no gross abnormalities. The appendix was obliterated, and the prostate was multinodular.

**Figure 2:**
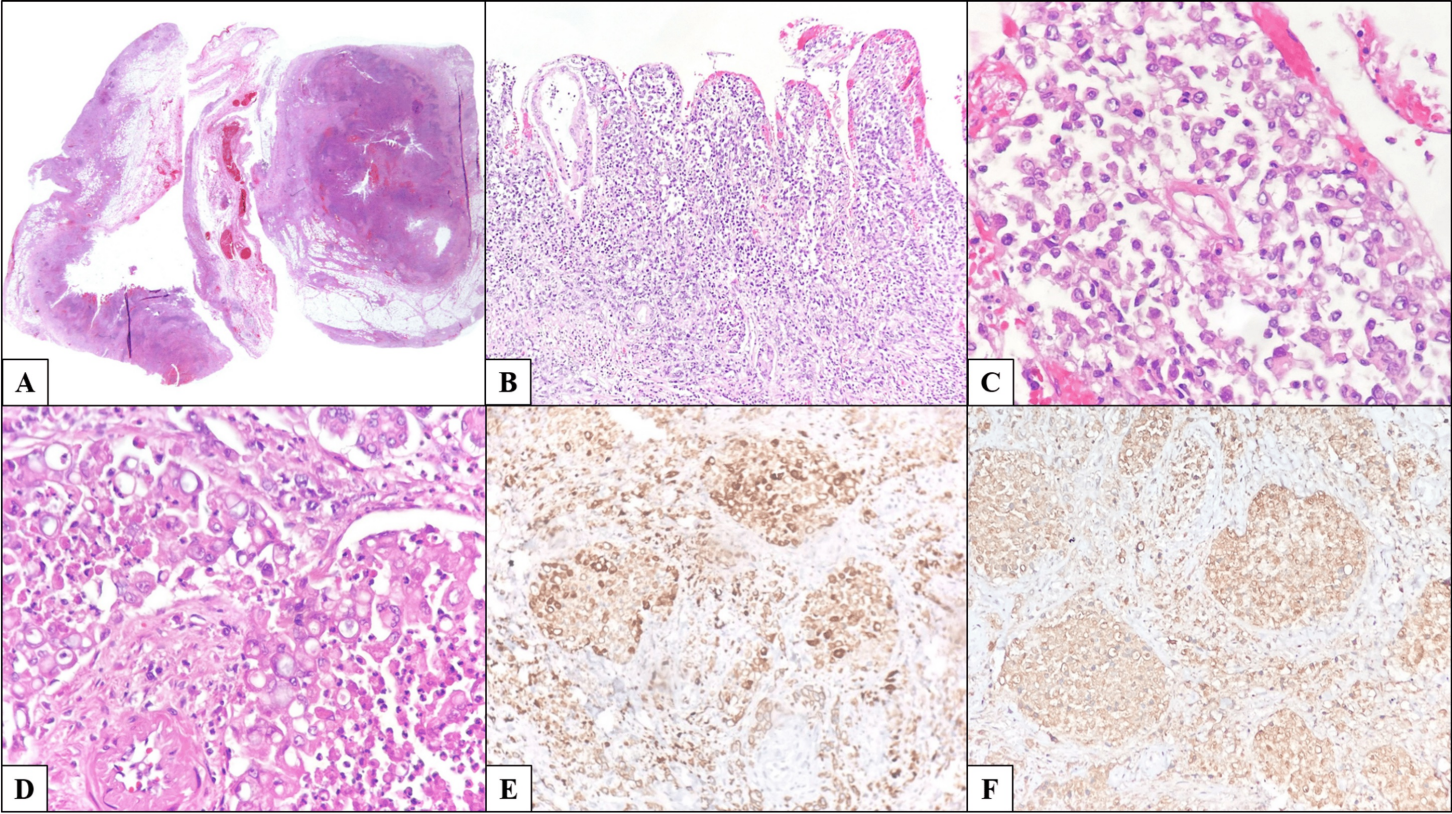
Histopathologic features of the primary tumor. (A) Low-power view showing diffuse thickening of the bile ducts by neoplastic infiltration with luminal narrowing (right), and a longitudinal section of the cystic duct with similar involvement (left). The central section corresponds to the gallbladder wall, which lacks epithelial involvement but shows lymphovascular invasion within the serosa (H&E, 20×). (B) Complete replacement of the biliary epithelium by neoplastic cells (H&E, 200×). (C) High-power view showing a diffuse growth pattern composed of round, hyperchromatic, atypical cells without evident differentiation (H&E, 400×). (D) Areas with abundant clear cytoplasm and signet ring-like morphology (H&E, 400×). (E) Tumor cells positive for CK19. (F) Tumor cells positive for carcinoembryonic antigen.

Microscopically, the lesion originated from the extrahepatic bile ducts and exhibited, where the greatest tumor burden was identified, completely replacing the ductal wall. No precursor lesion was identified despite extensive sampling. The tumor exhibited a solid and diffuse growth pattern with poorly defined infiltrative borders and extensive areas of necrosis. Tumor cells were round with hyperchromatic, pleomorphic nuclei; some showed abundant clear cytoplasm displacing the nuclei peripherally, imparting a signet ring-like appearance, with prominent perineural invasion and extensive lymphovascular invasion (Figure 2).

**Figure 3:**
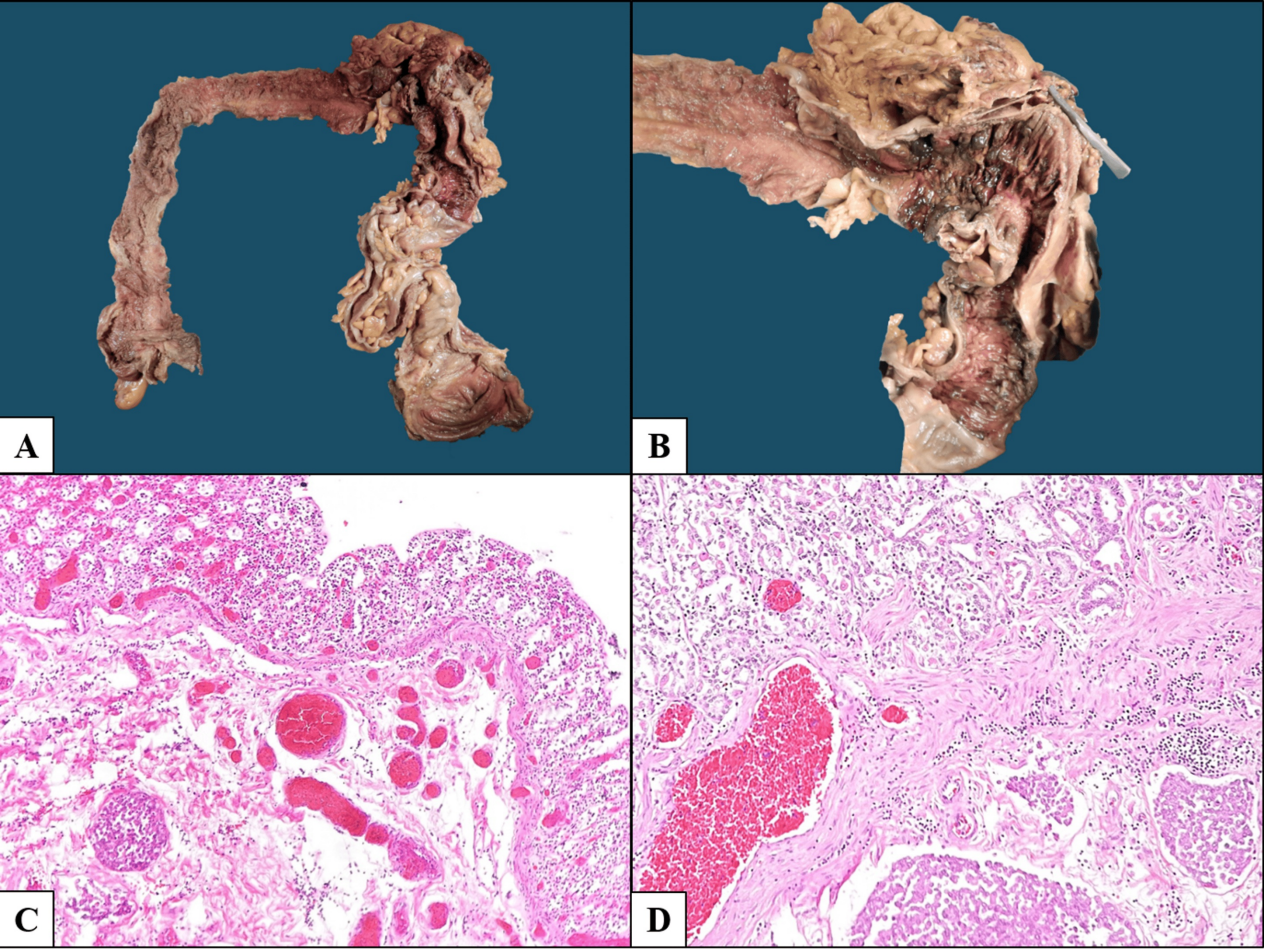
Gastrointestinal involvement. (A) Colon with direct neoplastic infiltration of the serosa of the transverse segment, with destruction of vascular structures. (B) Higher magnification showing mucosa distal to the infiltrated area with necrohemorrhagic changes secondary to ischemia. (C) Colonic wall with extensive ischemic mucosal injury and thrombosis of submucosal vessels (H&E, 200×). (D) Gastric wall showing ischemic mucosal changes secondary to vascular occlusion by neoplastic thrombi (H&E, 200×).

**Figure 4:**
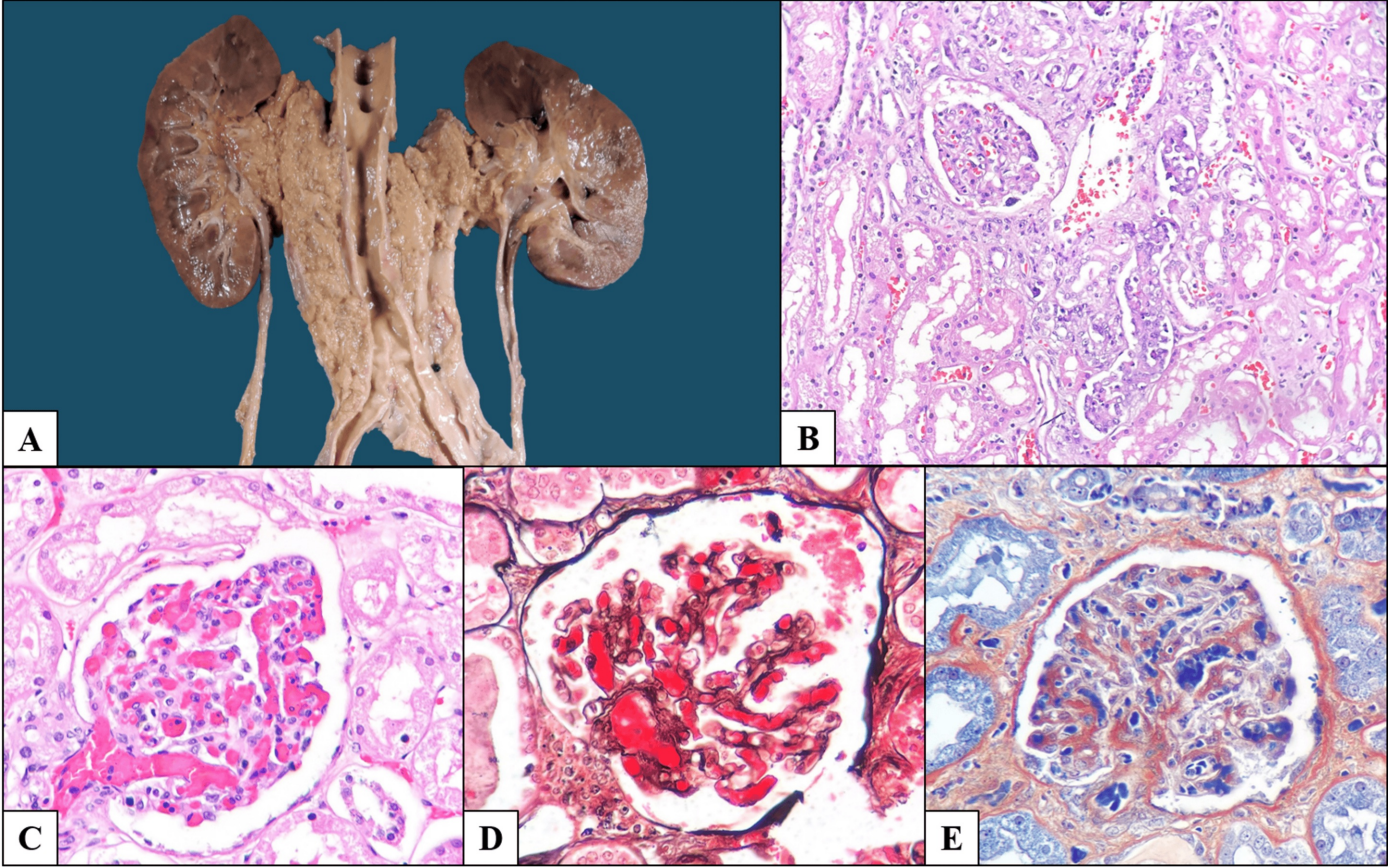
Renal involvement and thrombotic microangiopathy. (A) Renal block showing diffuse tumor infiltration of periaortic soft tissues with bilateral extension to the renal hila, resulting in right-sided hydronephrosis; the aorta shows atherosclerosis. (B) Neoplastic infiltration of the renal parenchyma (H&E, 200×). (C) Glomerulus with capillaries occluded by erythrocyte- and fibrin-rich thrombi (H&E, 400×). (D) Jones methenamine silver stain highlighting basement membranes and occlusive thrombi (400×). (E) Phosphotungstic acid hematoxylin stain demonstrating areas of fibrinoid necrosis (400×).

Organs involved macroscopically also demonstrated tumor infiltration microscopically, with areas of better differentiation showing irregular infiltrative gland formation (Figures 3, 4).

**Figure 5:**
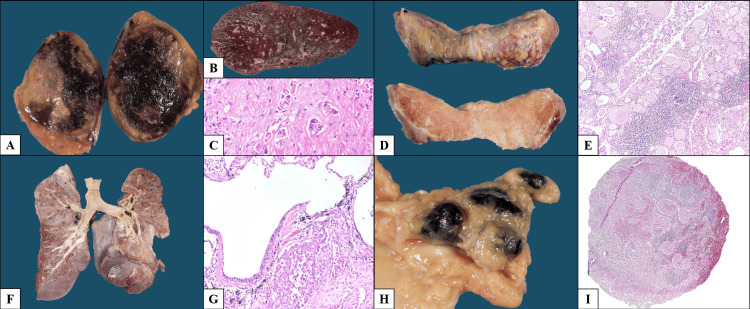
Multiorgan metastatic involvement. (A) Right adrenal gland with necrohemorrhagic metastasis. (B) Spleen with chronic passive congestion and no gross lesions. (C) Histologic section of the spleen showing neoplastic infiltration (H&E, 400×). (D) Thyroid gland with irregular external surface and smooth, opaque cut surface. (E) Diffuse infiltration of the thyroid by neoplastic cells with associated lymphoid aggregates, some with germinal centers (H&E, 200×). (F) Coronal section of the lungs without gross lesions. (G) Histologic section showing neoplastic infiltration of the pulmonary parenchyma (H&E, 200×). (H) Enlarged peritracheal lymph nodes with anthracosis. (I) Lymph node showing metastatic carcinoma in addition to anthracosis (H&E, 20×).

Additionally, microscopic involvement of the thyroid, lungs, spleen, and kidneys was identified, despite the absence of gross lesions (Figure 5).

Immunohistochemical studies showed tumor positivity for CK7, CK19, CK20, and carcinoembryonic antigen. Chromogranin and synaptophysin were negative. Mismatch repair proteins (MSH2, MSH6, MLH1, PMS2) were retained. Human Epidermal Growth Factor Receptor 2 (HER2) was negative. Additional stains, including Hepar-1, c-MYC, AML, and CD8, were negative. p53 showed a wild-type pattern, and the Ki-67 proliferation index was approximately 70%. Other findings included multiple myocardial calcifications, fibrosis of the atrioventricular node following dissection of the triangle of Koch, and atherosclerosis.

The final diagnosis was carcinoma of the extrahepatic bile ducts (perihilar extrahepatic cholangiocarcinoma) with multiorgan involvement. A comprehensive list of autopsy diagnoses and their clinicopathologic correlations is provided in Table 2.

**Table 2:** Final comprehensive pathologic diagnoses, ordered by causality. Table Credit: Javier A. Teco Cortes, Tania A. Galindo-García, Paola X. Calderón-Navarro

FINAL ANATOMOPATHOLOGICAL DIAGNOSES
Extrahepatic bile duct carcinoma (perihilar cholangiocarcinoma), originating in the cystic duct and common hepatic duct, poorly differentiated, with infiltration of the liver, pancreas, gastric wall, small intestine, colon, mesentery, periaortic soft tissues, and renal arterial and venous branches; bilateral renal hilum involvement; peritoneal and diaphragmatic carcinomatosis; metastases to thyroid, lungs, spleen, adrenal glands, and periaortic, peritracheal, and abdominal lymph nodes.
Proximal extrahepatic bile duct obstruction
Generalized jaundice
Ischemic-hemorrhagic cholecystitis
Multiple hepatic microabscesses
Regenerative hepatic parenchymal changes with intracellular cholestasis and ductular reaction
Bile pigment tubulopathy
Acute necrotizing pancreatitis
Infiltration of the portomesenteric axis and mesenteric artery-derived vessels
Predominantly antral ischemic gastropathy
Transmural ischemic colitis in the transverse colon
Ascites (1.5 L) and bilateral pleural effusions (right 300 mL, left 50 mL)
Bilateral proximal extrinsic ureteral obstruction, predominantly right-sided
Early right hydronephrosis
Multifocal acute tubular injury with moderate regenerative epithelial changes
Acute thrombotic microangiopathy in kidneys
Advanced calcified atherosclerosis, mainly affecting the abdominal aorta and coronary arteries
Chronic ischemic cardiomyopathy
Fibromyxoid degeneration of cardiac valves
Multiple intramyocardial calcifications and cytoplasmic lipofuscin deposits
Fibrosis of the atrioventricular conduction node (history of atrial flutter)
Grade 2 macrovesicular hepatic steatosis
Focal segmental glomerulosclerosis (maladaptive type)
Grade 1 renal interstitial fibrosis
Chronic lymphocytic thyroiditis
Pulmonary anthracosis with bilateral peribronchial and peritracheal lymph node involvement
Appendiceal neuroma
Fibroglandular prostatic hyperplasia
Testicular atrophy

## Discussion

Extrahepatic bile duct carcinoma is an uncommon neoplasm that most frequently presents with obstructive jaundice; however, it may manifest with a wide spectrum of clinical features. When combined with its anatomically challenging location for biopsy, early diagnosis may be particularly difficult, as illustrated by the present case.

Our patient exhibited classic features of extrahepatic cholangiocarcinoma, including jaundice, secondary hepatic injury due to biliary obstruction, and pancreatic infiltration with necrosis. Nevertheless, the case was further complicated by pleural effusions, ascites, lower gastrointestinal bleeding, ischemic gastropathy, hydronephrosis, and acute kidney injury with acute thrombotic microangiopathy. The autopsy allowed not only documentation of these findings but also a clear demonstration of their causal relationship with the underlying neoplasm. 

The absence of biliary dilation on imaging despite large duct obstruction at autopsy may be explained by a predominantly infiltrative growth pattern with periductal spread, which can restrict ductal expansion. Additionally, extensive extrahepatic tumor burden may have limited upstream dilation. Rapid clinical deterioration may also have contributed to the discrepancy between imaging and autopsy findings.

Lower gastrointestinal bleeding has not been previously reported as a direct manifestation of cholangiocarcinoma. The only related report described upper gastrointestinal bleeding secondary to radiofrequency ablation-related gastric perforation in intrahepatic cholangiocarcinoma [5]. In contrast, our case demonstrated that the gastrointestinal bleeding resulted directly from neoplastic infiltration of the colonic wall, with destruction of the mesenteric vascular supply, leading to ischemic necrosis and mucosal hemorrhage.

Although thrombotic microangiopathy (TMA) has been reported in association with cholangiocarcinoma, previously described cases involved metastatic disease treated with gemcitabine [6,7]. In our patient, no prior therapy had been administered, supporting a direct causal relationship between the neoplasm and endothelial injury, consistent with reports in other malignancies [8]. Proposed mechanisms include direct tissue invasion, tumor-associated neoangiogenesis, tumor necrosis and degradation, disruption of endothelial integrity, tumor-induced hypoxia, and activation of the hemostatic system [9].

Acute kidney injury has been reported in perihilar cholangiocarcinoma due to rapid biliary drainage [10]; in our case, renal injury was attributable to both direct parenchymal infiltration by the tumor and hydronephrosis caused by extrinsic ureteral compression at the renal hilum. Given the severe hyperbilirubinemia, bile cast nephropathy may have contributed a secondary factor to the patient’s acute kidney injury.

Immunohistochemical evaluation for microsatellite instability and HER2 overexpression was performed and yielded negative results. Nevertheless, routine assessment of these markers is currently recommended, as identification of microsatellite instability or HER2 overexpression may guide targeted therapies. HER2 overexpression, although rare in intrahepatic cholangiocarcinoma, has been reported in up to 19.9% of extrahepatic cases [11], making anti-HER2 therapies a potential treatment option [12]. Likewise, microsatellite instability cholangiocarcinomas exhibit higher tumor mutational burden and increased PD-L1 expression, and patients treated with PD-1 inhibitors demonstrate improved overall and progression-free survival [13,14]. Tumor heterogeneity should also be considered, as discordant microsatellite status between primary and metastatic sites has been reported [15].

The presence of a neuroendocrine component in extrahepatic cholangiocarcinoma has been associated with more advanced disease and increased metastatic potential, yet paradoxically with improved overall survival [16]. In our case, chromogranin and synaptophysin were negative, excluding this component.

Montal et al. proposed a molecular classification of extrahepatic cholangiocarcinoma into four classes, metabolic, proliferative, mesenchymal, and immune, based on genomic alterations [17]. Although molecular testing was not available in our case, immunohistochemistry was used as a surrogate approach; however, negative results precluded assignment to a specific molecular class, underscoring the limitations of immunohistochemistry as a substitute for molecular profiling. Importantly, this molecular classification has not yet demonstrated a significant impact on treatment or prognosis, with tumor stage at diagnosis remaining the most critical prognostic factor.

The autopsy not only confirmed the classic association between cholangiocarcinoma and biliary obstruction but also unequivocally demonstrated the relationship between diffuse tumor infiltration and the patient’s atypical clinical manifestations. Necrohemorrhagic colitis, ischemic gastropathy, ascites, and pleural effusions were explained by vascular infiltration and destruction of branches of the superior and inferior mesenteric arteries, leading to impaired perfusion and increased intravascular hydrostatic pressure. Hydronephrosis resulted from direct tumor infiltration of the renal hilum, and TMA was attributable to the tumor-induced procoagulant state. This case underscores the enduring value of autopsy studies in modern medicine, prompting reflection on the limitations of partial clinical assessment and reinforcing the importance of comprehensive pathologic evaluation.

## Conclusions

Extrahepatic cholangiocarcinoma is a highly aggressive malignancy arising from the extrahepatic bile ducts, with a poor prognosis and near-zero survival in unresectable disease. Early diagnosis is crucial to allow potentially curative treatment; however, nonspecific symptoms, difficult biopsy access, and atypical clinical manifestations often delay diagnosis. This case highlights a broad spectrum of both typical and atypical manifestations that should be considered in clinical practice, as failure to recognize them may contribute to diagnostic delay and adversely affect patient outcomes.
